# Structural Damage Identification Based on Integrated Utilization of Inverse Finite Element Method and Pseudo-Excitation Approach

**DOI:** 10.3390/s21020606

**Published:** 2021-01-16

**Authors:** Tengteng Li, Maosen Cao, Jianle Li, Lei Yang, Hao Xu, Zhanjun Wu

**Affiliations:** 1 State Key Laboratory of Structural Analysis for Industrial Equipment, School of Aeronautics and Astronautics, Faculty of Vehicle Engineering and Mechanics, Dalian University of Technology, Dalian 116024, China; litengteng@mail.dlut.edu.cn (T.L.); LiJianle@mail.dlut.edu.cn (J.L.); yangl@dlut.edu.cn (L.Y.); wuzhj@dlut.edu.cn (Z.W.); 2 Department of Engineering Mechanics, Hohai University, Nanjing 210098, China; cmszhy@hhu.edu.cn

**Keywords:** structural health monitoring, damage identification, inverse finite element method, pseudo-excitation approach, structural vibration

## Abstract

The attempt to integrate the applications of conventional structural deformation reconstruction strategies and vibration-based damage identification methods is made in this study, where, more specifically, the inverse finite element method (iFEM) and pseudo-excitation approach (PE) are combined for the first time, to give rise to a novel structural health monitoring (SHM) framework showing various advantages, particularly in aspects of enhanced adaptability and robustness. As the key component of the method, the inverse finite element method (iFEM) enables precise reconstruction of vibration displacements based on measured dynamic strains, which, as compared to displacement measurement, is much more adaptable to existing on-board SHM systems in engineering practice. The PE, on the other hand, is applied subsequently, relying on the reconstructed displacements for the identification of structural damage. Delamination zones in a carbon fibre reinforced plastic (CFRP) laminate are identified using the developed method. As demonstrated by the damage detection results, the iFEM-PE method possesses apparently improved accuracy and significantly enhanced noise immunity compared to the original PE approach depending on displacement measurement. Extensive parametric study is conducted to discuss the influence of a variety of factors on the effectiveness and accuracy of damage identification, including the influence of damage size and position, measurement density, sensor layout, vibration frequency and noise level. It is found that different factors are highly correlated and thus should be considered comprehensively to achieve optimal detection results. The application of the iFEM-PE method is extended to better adapt to the structural operational state, where multiple groups of vibration responses within a wide frequency band are used. Hybrid data fusion is applied to process the damage index (DI) constructed based on the multiple responses, leading to detection results capable of indicating delamination positions precisely.

## 1. Introduction

In recent decades, structural health monitoring (SHM) has undergone rapid development as an interdisciplinary area embracing a variety of theories, methods and techniques [1,2,3,4]. When applied in different structural types (i.e., beams, plates, shells, etc.), most SHM techniques aim at acquiring information about the damage and/or state quantities of structures. While studies on the former category have attracted extensive interest [5,6,7], as damage information is no doubt a direct sign reflecting the health condition of structures, the latter, on the other hand, deserves at least equal attention by noticing that state quantities, such as deformed shapes [8,9,10,11], strain/stress distributions, external loads [12,13], vibration characteristics [14,15,16], etc., are among the root causes of damage occurrence and evolution, and are also responsible for various non-damage-related structural failures such as buckling. Therefore, to guarantee an accurate estimation of structural health condition, the complete picture of SHM, including both damage and state sensing, should always be kept in consideration by scholars and engineers.

Among various state sensing methods, the inverse finite element method (iFEM) has been demonstrated to be promising in reconstructing full-filed displacements in different structures [17,18,19]. Inheriting the strategy of geometric discretization used by direct FEM, iFEM is featured by a least-square function consisting of analytical and measured strains, and the minimization of the functional results in the solutions of structural displacements. To date, several versions of inverse elements have been developed [20,21,22,23], and the effectiveness of these versions has been examined in a variety of structures, in particular those with complex geometries [24,25]. Recently, efforts have been made to explore the potential of iFEM in damage identification. Cracks are numerically identified in a plate structure using a damage index (DI) established based on the difference between the strains measured on the damaged structure and the baseline strains reconstructed by iFEM [26]. Dent damage in a numerical stiffened cylindrical structure is detected in a similar way, whereas the only difference is that the baseline strains are captured from an undamaged benchmark structure [27]. Different from FE model updating, which is an off-line iterative process with damage built and updated in the FE model, iFEM is an on-line non-iterative process, where damage is identified according to the features of the reconstructed structural responses.

While the iFEM has shown certain sensitivity to damage, the current form of DI, essentially depending on local strain variation before and after damage occurrence, is limited in the accuracy of damage identification. One reason is that the area of local strain variation caused by damage (e.g., cracks, delaminations, etc.) is often much larger in size than the actual damaged zone, and hence the size and shape of the damaged zone cannot be revealed merely according to strain variation; another reason is that damage, although small in size, may cause global variation of structural responses (e.g., deformed shapes). As a consequence, the baseline signal obtained using iFEM, either directly from a damaged structure or an intact benchmark structure, is not able to generate an ideal DI which is only sensitive to the damaged zone. In other words, the DI may show non-zero fluctuations in the undamaged regions of the structure, which may degrade the damage identification accuracy or even lead to false alarms at the undamaged regions. Such a phenomenon would be obvious under structural vibration states, where the displacement/strain fields exhibit more complex spatial alternation.

Vibration-based methods have been widely applied in damage identification for decades [28,29,30,31]. Compared with static structural response, vibration responses contain much more ‘local’ features that can be linked with damage. A method named pseudo-excitation (PE) was proposed recently for quantitative damage detection [32,33,34,35,36,37]. The principle of this approach relies on point-to-point examination of dynamic equilibrium conditions of structural components (e.g., beams, plates, etc.). Established based on equations of motion, the DI of the PE approach possesses a solid physical implication, and is in theory able to recognize not only the location but also the shape of small damage, where the boundaries of damaged zones can be depicted precisely. In addition, this approach also has some unique advantages, such as independence of baseline signals and benchmark structures, which is of importance for online damage identification. The spreading of PE in applications, however, is limited due to two major obstacles. The first one is that as an equation of motion is directly used, the approach depends on the measurement of vibration displacements. Under laboratory conditions, vibration displacements can be measured precisely using devices such as a scanning laser vibrometer [34]. For operational structures, however, dense displacement measurement over large areas is extremely challenging. The other obstacle is the large vulnerability of the approach to measurement noise. Specifically, the noise in displacements will be amplified drastically by the high-order derivative terms in the DI formulation, resulting in seriously reduced accuracy of damage identification.

The integrated implementation of the iFEM and PE approach is able to achieve several unique advantages. By using iFEM, strain signals can be transformed into full-filed displacements, used then as inputs of the PE approach to construct its DI. That means, with greater dependence on displacement measurement, the PE approach can be applied by using various types of strain sensors, which can be easily integrated into operational structures and on-board SHM systems. Moreover, the measurement of strains could largely suppress the interference of noise on the DI, mainly due to the alleviated noise amplification effect associated with the high-order derivatives. Finally, the accuracy of the original iFEM in identifying damage is largely enhanced by integrating the PE approach, the DI of which exhibits singularity within the damaged zone (especially at the damage boundaries), whereas it is ideally zero in the undamaged areas.

A challenge of implementing the iFEM-PE method might be the demand of high measurement density requiring a large number of strain sensors. Along with the rapid advancement of sensor technology, however, more and more high-density, structure-friendly sensor networks have been developed. For example, a high-density, graphite-based strain sensor array was screen printed with the shortest distance between adjacent sensors of 9 mm [38]. The sensor array was used to measure the dynamic strain responses of a highway bridge. In addition, a variety of lightweight, flexible nanocomposite sensors have been fabricated. The response frequencies of certain types of nanocomposites are able to reach several hundreds of kilohertz, which is sufficient in capturing structural vibration characteristics [39,40,41]. Moreover, extensive investigations have focused on lightweight fiber-optic sensor arrays in recent years. Being analogous to human neural system, ultra-thin optical fibers can be embedded in target structures to sense strain variations within a large spatial range. The fiber Bragg grating (FBG) sensor network has already become a common option in SHM, thanking to its inclusion of a large number of sensors and its capacity for a fast response [42,43,44]. On the other hand, distributed optical fiber sensors are receiving increasing attention due to their appealing potential in dense measurement of strains [45,46,47]. The number of measurement points can easily reach the hundreds or even thousands by using a single distributed optical fiber, and the response frequencies of these sensors are under rapid yearly increase.

In this study, the iFEM and PE approaches are integrated for the first time to perform real-time damage identification based on structural vibration responses. The strategy of sensor layout and grid mapping is established to ensure efficient data transfer from the iFEM to the PE approach. The accuracy and noise immunity of the iFEM-PE method are examined by identifying multiple delamination zones in a CFRP laminate. Extensive parametric discussion is undertaken to explore the influence of a variety of factors on the accuracy of damage detection. The factors include damage sizes and positions, measurement densities, way of sensor layouts, vibration frequencies, noise influence. Ultimately, a hybrid data fusion scheme is applied to enhance the robustness of the method in processing multiple groups of vibration signals within a wide frequency band.

## 2. Methodologies

### 2.1. The Inverse Finite Element Method (iFEM)

The iFEM is implemented by first discretizing the geometry of the inspected structure using a number of inverse elements. In this study, a recently developed quadrilateral inverse shell element, iQS4, is adopted due to its satisfactory performance in dealing with shell structures and membrane problems by virtue of the inclusion of drilling rotations [23,24,25].

#### 2.1.1. Kinematic Relations

**Figure 1:**
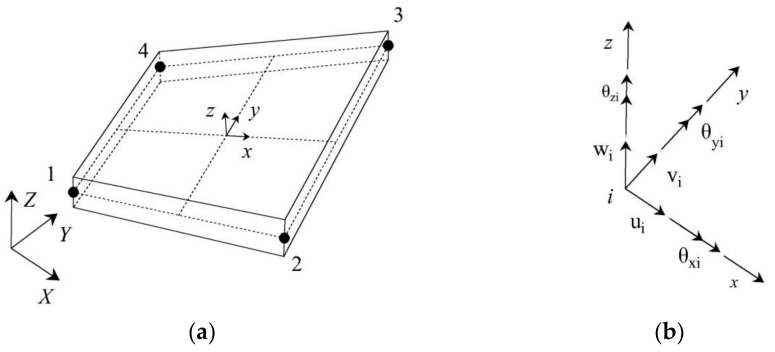
(**a**) The iQS4 inverse element and its (**b**) local nodal degrees of freedom.

An arbitrary four-node iQS4 element of thickness 2 h is shown in Figure 1a. According to Mindlin plate theory, there are six degrees-of-freedom (DOFs) at each node, as shown in Figure 1b, where u and v are in plane translations; w is transverse deflection; $\theta_{x}$ and $\theta_{y}$ are bending rotations, and $\theta_{z}$ is in-plane rotation.

The generalized strains are defined to include eight components, i.e.,

(1) $$\left\{\varepsilon_{1},\varepsilon_{2},\ldots \varepsilon_{8}\right\}={\left\{e,\kappa ,g\right\}}^{T}$$

where **e**, κ and **g** represent membrane strains, bending curvatures and transverse shear strains, respectively, which are expressed by

(2) $$e=\left[\begin{array}{ccc} \varepsilon_{1} & \varepsilon_{2} & \varepsilon_{3} \end{array}\right]=\left[\begin{array}{ccc} B_{1}u^{e} & B_{2}u^{e} & B_{3}u^{e} \end{array}\right]$$

(3) $$\kappa =\left[\begin{array}{ccc} \varepsilon_{4} & \varepsilon_{5} & \varepsilon_{6} \end{array}\right]=\left[\begin{array}{ccc} B_{4}u^{e} & B_{5}u^{e} & B_{6}u^{e} \end{array}\right]$$

(4) $$g=\left[\begin{array}{cc} \varepsilon_{7} & \varepsilon_{8} \end{array}\right]=\left[\begin{array}{cc} B_{7}u^{e} & B_{8}u^{e} \end{array}\right]$$

where $u^{e}={\left[\begin{array}{cccc} u_{1}^{e} & u_{2}^{e} & u_{3}^{e} & u_{4}^{e} \end{array}\right]}^{T}$ represents all nodal DOFs of the iQS4 element, with $u_{i}^{e}={\left[\begin{array}{cccccc} u_{i} & v_{i} & w_{i} & \theta_{xi} & \theta_{yi} & \theta_{zi} \end{array}\right]}^{T}(i=1,\ldots ,4)$ denoting the six DOFs at a particular node. The matrices $B_{i}\;(i=1\cdots 8)$ contain derivatives of the shape functionals, as detailed in [1].

#### 2.1.2. In Situ Strain Measurement

**Figure 2:**
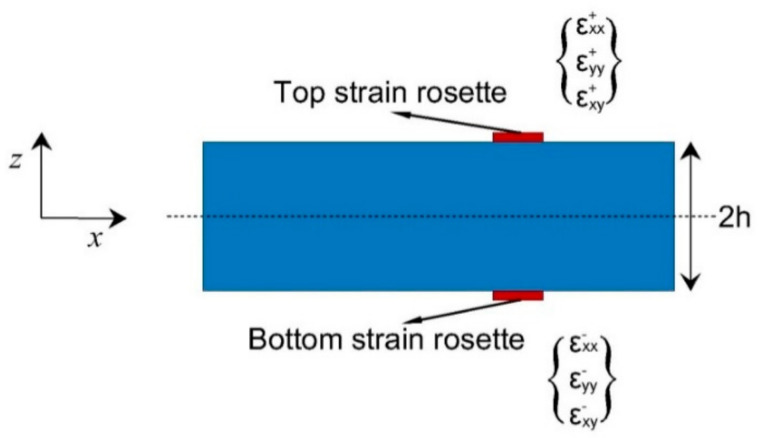
Discrete strains measured on structural surface.

As the counterparts of the analytical strains shown in Equations (3) and (4), strains are measured experimentally on the upper (+) and lower (−) surfaces of a plate, respectively, as illustrated in Figure 2. At a particular in-plane position, the membrane strains and bending curvatures can be constructed according to

(5) $$e_{i}^{\varepsilon }\equiv {\left\{\begin{array}{c} \varepsilon_{1}^{\varepsilon }\\ \varepsilon_{2}^{\varepsilon }\\ \varepsilon_{3}^{\varepsilon } \end{array}\right\}}_{i}=\frac{1}{2}{\left\{\begin{array}{c} \varepsilon_{xx}^{+}+\varepsilon_{xx}^{-}\\ \varepsilon_{yy}^{+}+\varepsilon_{yy}^{-}\\ \gamma_{xy}^{+}+\gamma_{xy}^{-} \end{array}\right\}}_{i}$$

and

(6) $$\kappa_{i}^{\varepsilon }\equiv {\left\{\begin{array}{c} \varepsilon_{4}^{\varepsilon }\\ \varepsilon_{5}^{\varepsilon }\\ \varepsilon_{6}^{\varepsilon } \end{array}\right\}}_{i}=\frac{1}{2h}{\left\{\begin{array}{c} \varepsilon_{xx}^{+}-\varepsilon_{xx}^{-}\\ \varepsilon_{yy}^{+}-\varepsilon_{yy}^{-}\\ \gamma_{xy}^{+}-\gamma_{xy}^{-} \end{array}\right\}}_{i}$$

where the measured surface strains are denoted by ${\left\{\begin{array}{ccc} \varepsilon_{xx}^{+} & \varepsilon_{yy}^{+} & \gamma_{xy}^{+} \end{array}\right\}}_{i}$ and ${\left\{\begin{array}{ccc} \varepsilon_{xx}^{-} & \varepsilon_{yy}^{-} & \gamma_{xy}^{-} \end{array}\right\}}_{i}$; the superscript ε implies the existence of errors involved in strain measurement. In application, the surface strains can be measured by sensors such as conventional strain gauges or embedded fiber-optic networks. The measurement of the transverse shear strain, $g^{\varepsilon }$, is often non-trivial, where multiple layers of sensors along the plate thickness are often required. However, the magnitudes of transverse shear strains are normally insignificant particularly in thin-walled structures. Thus the experimental part of the transverse shear strains can be omitted. Moreover, the in-plane shear strain, i.e., $\gamma_{xy}^{+}$ and $\gamma_{xy}^{-}$ in Equations (5) and (6), are also omitted due to their insignificant magnitudes in the present work. Therefore, only linear strains are utilized for displacement reconstruction in the following study.

#### 2.1.3. Establishment of the Weighted Least-Square Functional

By using the analytical and experimental strains as presented in Equations (2)–(6), respectively, a weighted least-square functional is established. For an iQS4 element, the functional, $\Phi_{e}(u^{e})$, is defined as [2]

(7) $$\Phi_{e}(u^{e})=\sum_{k=1}^{8}w_{k}{\left\|\varepsilon_{k}(u^{e})-\varepsilon_{k}^{\varepsilon }\right\|}^{2}$$

where $\varepsilon_{k}(u^{e})$ and $\varepsilon_{k}^{\varepsilon }$ represent the analytical and experimental strains at a given point, respectively. The squared norm in the equation is written in terms of a normalized Euclidean norm, as

(8) $$\left\|\varepsilon_{k}(u^{e})-\varepsilon_{k}^{\varepsilon }\right\|=\frac{1}{n}\int_{A^{e}}\sum_{i=1}^{n}{\left[\varepsilon_{k(i)}(u^{e})-\varepsilon_{k(i)}^{\varepsilon }\right]}^{2}dA^{e}$$

where $A^{e}$ represents the mid-plane area of the element and n is the number of sensors located in the element.$w_{k}$ represents positive weighting constants which determine the extent of constraint between the analytical strains and its experimental counterparts. Generally, it has $\left\{w_{k}\}=\{\lambda_{1},\lambda_{2},4h^{2}\lambda_{3},4h^{2}\lambda_{4},4h^{2}\lambda_{5},\lambda_{6},\lambda_{7},\lambda_{8}\right\}$. If all measured values in $\left\{\varepsilon^{\varepsilon }\right\}$ are available, the weighting coefficients are set as $\lambda_{k}=1$(k=1,⋯,8), but if certain strain terms are omitted, the corresponding weighting coefficients should be set to be significantly small, e.g., $\lambda_{k}=10^{-4}$.

Once the least-square functional is established for each element, the functional is minimized with respect to the nodal DOFs, resulting in a series of equations:

(9) $$\frac{\partial \Phi_{e}(u^{e})}{\partial u^{e}}=k^{e}u^{e}-f^{e}=0\Rightarrow k^{e}u^{e}=f^{e}$$

where $k^{e}$ is the element left-hand-side matrix, and $f^{e}$ is the element right-hand-side vector that is a functional of the measured strain values. Then, the local matrices are assembled into a global matrix, which can be feasibly solved to obtain the displacement field of target structure.

### 2.2. Pseudo-Excitation (PE) Approach

The damage index, DI, of the PE approach is established based on equations of motions of different types of structural components. For an isotropic plate component, DI can be expressed as

(10) $$\mathrm{DI}\left(x,y\right)=D\left(\frac{\partial^{4}w\left(x,y\right)}{\partial x^{4}}+2\frac{\partial^{4}w\left(x,y\right)}{\partial x^{2}\partial y^{2}}+\frac{\partial^{4}w\left(x,y\right)}{\partial y^{4}}\right)-\rho h\omega^{2}w\left(x,y\right)$$

where *D* is the bending stiffness; $w\left(x,y\right)$ is vibration displacement; and ρ, *h* and ω are the density, thickness and angular vibration frequency, respectively. Since composite laminates are investigated in this study, the DI is constructed according to laminate vibration theory, leading to a more complex form as

(11) $$\mathrm{DI}\left(x,y\right)=D_{11}\frac{\partial^{4}w\left(x,y\right)}{\partial x^{4}}+4D_{16}\frac{\partial^{4}w\left(x,y\right)}{\partial x^{3}\partial y}+2(D_{12}+2D_{66})\frac{\partial^{4}w\left(x,y\right)}{\partial x^{2}\partial y^{2}}\;+4D_{26}\frac{\partial^{4}w\left(x,y\right)}{\partial x\partial y^{3}}+D_{22}\frac{\partial^{4}w\left(x,y\right)}{\partial y^{4}}-\rho h\omega^{2}w\left(x,y\right)$$

where $D_{ij}$ is the bending stiffness matrices of the laminate. In both Equations (10) and (11), it can be realized that when considering elemental equilibrium condition, the right-hand side is the combination of the internal and inertia forces, whereas the left-hand side is the external excitation. As demonstrated in [34], the values of right-hand side at areas without external excitation but containing damage will show prominent singularities at the damaged zones. Such singularities can also be observed in DI caused by excitations. Therefore, damage is deemed as certain kind of ‘pseudo’ excitation (PE). At areas without either damage or excitations, the DI values are strictly zero due to the satisfaction of equilibrium condition. Such a property of DI is ideal for damage indication.

In applications, the construction of DI relies on the measurement of vibration displacements, which can be captured densely using equipment such as a scanning laser vibrometer (SLV). Specifically, vibration displacements were measured at points of a two-dimensional grids pre-assigned on the plate surface, and the high order derivatives of displacements, as included in Equation (11), are calculated according to finite difference scheme [34]. Although exhibiting significant accuracy in the quantitative identification of small damage, the PE approach is highly vulnerable to measurement noise, as will be explained later.

### 2.3. The iFEM-PE Method

**Figure 3:**
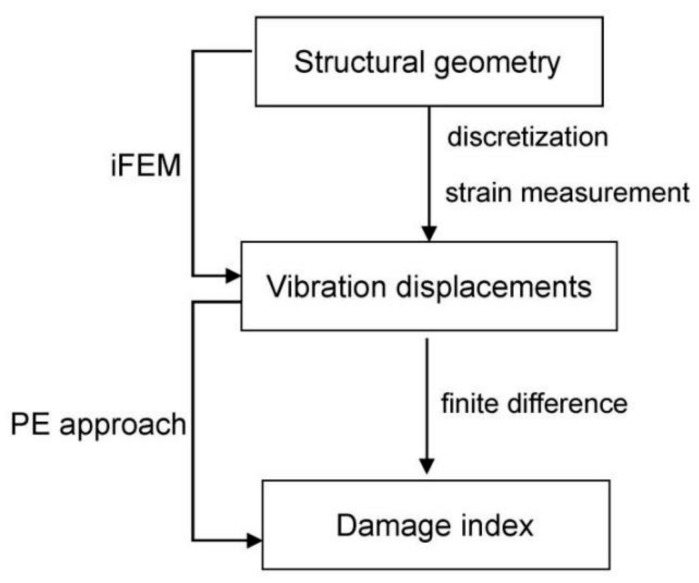
Flowchart of the inverse finite element method and pseudo-excitation (iFEM-PE) method.

**Figure 4:**
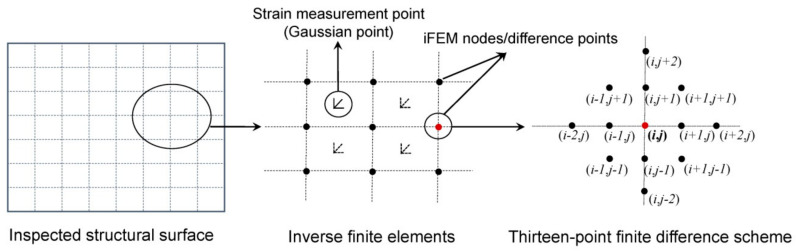
Point mapping and finite difference schemes for the iFEM-PE method.

The flowchart of the iFEM-PE method is shown in Figure 3. The iFEM is able to rapidly transform measured dynamic strains into vibration displacements, used then as inputs of the PE approach. Specifically, the nodes of the inverse elements of iFEM need to be mapped to the difference points of PE. A straightforward way is to enable a full coincidence between the iFEM nodes and the difference points, as illustrated by Figure 4, where the difference points of PE in a thirteen-point finite difference scheme are also shown [34]. It should be noted that the strain measurement points are located at the Gauss integration points of the inverse elements rather than at the nodes of the elements.

## 3. Damage Identification in a CFRP Laminate Using the iFEM-PE Method

### 3.1. Numerical Model

**Figure 5:**
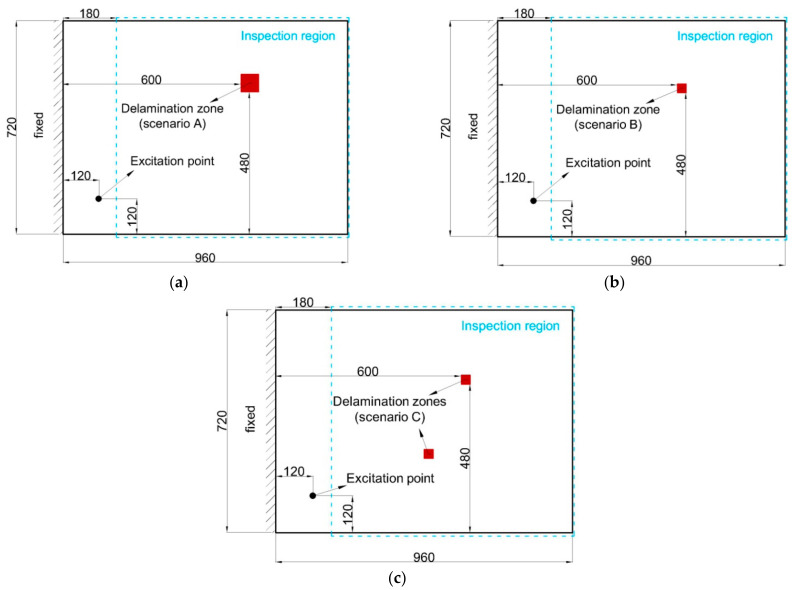
The dimensions of the laminate and delaminations in scenarios (**a**) A, (**b**) B and (**c**) C.

The effectiveness of the iFEM-PE method is verified using a cantilever CFRP laminate subject to different damage scenarios, as shown in Figure 5a–c. The laminate is made of 20 layers of unidirectional lamina with a symmetric stacking sequence of [0_2_/-45_2_/45_2_/90_2_/0_2_]s. The material properties of an individual lamina are listed in Table 1. A critical damping fraction of 5% is assigned. The width and length of the laminate are 720 and 960 mm, respectively, and the thickness of each lamina is 0.15 mm, giving rise to a total thickness of 3 mm.

Delaminations, as the most common damage form in composite structures, are embedded in the laminate. Three different delamination scenarios (denoted as A, B and C) are created. The first two include single damaged zones, where A corresponds to a relatively large damage size (measuring $60\times 60\;{\mathrm{mm}}^{2}$) and B corresponds to a small size (measuring $30\times 30\;{\mathrm{mm}}^{2}$). Scenario C, on the other hand, contains two small delamination zones each measuring $30\times 30\;{\mathrm{mm}}^{2}$. All delaminations were embedded at the 0/0 interface of the laminate. The dimension of the laminate and damage are presented in Figure 5a–c.

High-fidelity FEM analysis was first carried out using ABAQUS^®^ commercial code to generate strain data that were measured in experiments. The cantilever laminate was uniformly meshed by small S4R shell elements with dimensions of $1.5\times 1.5\;{\mathrm{mm}}^{2}$, based on which significantly dense strain data were obtained. A point-force perpendicular to the plate was applied to generate harmonic excitations. The excitation frequency was selected to be 200 Hz. To simulate the delaminations, an adhesive layer was built at the interface where the delaminations were located. The layer was modeled with isotropic material with Young’s moduli of 7 GPa, Poisson’s ratio of 0.3 and density of 1160 ${\mathrm{kg}/m}^{3}$. The delaminations were simulated by reducing the Young’s moduli of the adhesive layer to be 0.1 MPa.

### 3.2. Reconstruction of Vibration Displacements under Intact State

iFEM was applied based on square-shape iQS4 elements by using bi-axial, linear strains extracted from direct FEM. Relatively dense inverse elements with side length of 3mm were adopted to provide accurate results, and the surface strains were extracted at the Gaussian points of the inverse elements. Since the deformations of the laminate are mainly associated with bending, strains only on one side of the laminate (i.e., the top surface) were used, whereas strains on the other side (i.e., the bottom surface) were derived indirectly. In the current case, such derivation was straightforward, since strains on the opposite sides are assumed to have opposite signs. Such a treatment can largely reduce the amount of strain sensors needed in experiments.

The vibration displacements of the laminate under its intact state, i.e., without damage, were reconstructed using iFEM. To simulate the influence of measurement noise due to factors such as measurement errors, following equation is used

(12) $$\varepsilon^{\mathrm{noisy}}=\varepsilon^{\mathrm{exact}}\left(1+\Delta r\right)$$

where $\varepsilon^{\mathrm{noisy}}$ and $\varepsilon^{\mathrm{exact}}$ are strain data with and without noise influence, respectively. Δr is a Gaussian random number, the mean of which is zero and the standard derivation of which, $\sigma \left(\Delta r\right)$, represents the level of noise influence.

**Figure 6:**
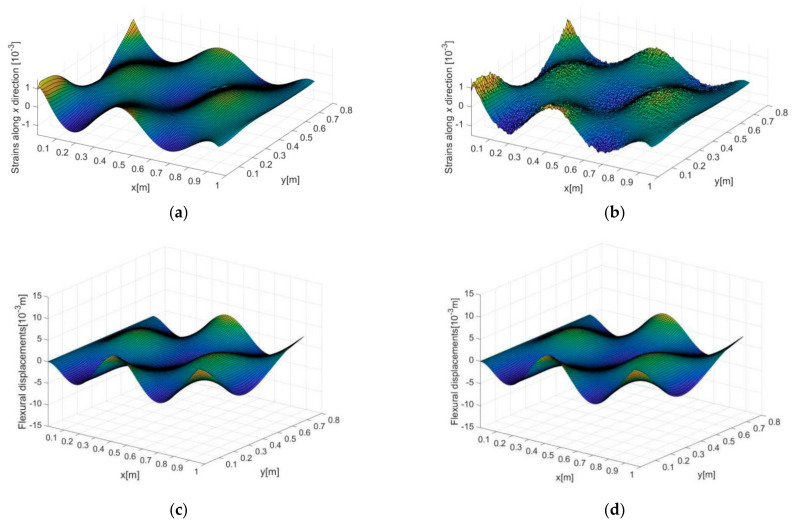
The strain distribution in the x direction (**a**) computed by direct FEM and then (**b**) contaminated using Equation (6) with noise level of 10%; the vibration displacements (**c**) computed by direct FEM and (**d**) reconstructed by iFEM based on strains in (**b**). The excitation frequency and size of the inverse elements are 200 Hz and 3 mm, respectively.

Figure 6a–d present the distributions of dynamic strains and vibration displacements. The exact strain distribution along x direction computed using direct FEM is presented in Figure 6a (strains along y direction are not shown), and measurement noise with level of 10%, i.e., $\sigma \left(\Delta r\right)=10\%$, is added to the strains according to Equation (12), as shown in Figure 6b. In the figure, a large extent of noise interference can be seen according to the drastic local disturbances of the strains. The exact displacements extracted from direct FEM and the reconstructed ones using iFEM based on the noisy strains (in Figure 6b) are presented in Figure 6c,d, respectively. Remarkable immunity to noise interference of the reconstructed displacements can be observed, where no obvious signal disturbances in the displacements are found in Figure 6d compared to that in Figure 6c.

Such observation demonstrates the advantage of iFEM in displacement reconstruction. An explanation is that as displacements are integrations of strains in essence, the local disturbances in strains are ‘smoothed’ during the reconstruction (integration) process. However, such an advantage tends to be less effective in damage identification, since damage-induced signal features are also with natures of local disturbances, which should be preserved as complete as possible during the reconstruction process. Therefore, more complicated factors should be considered for damage detection than for displacement reconstruction.

**Figure 7:**
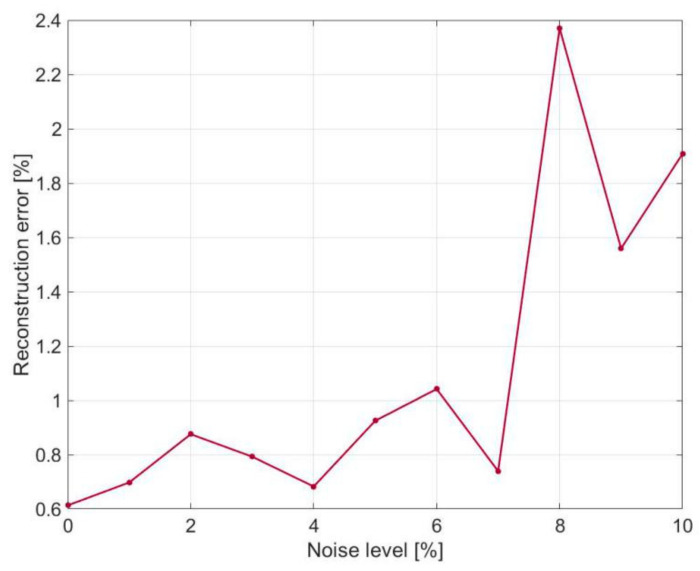
Errors of the reconstructed vibration displacements subject to different levels of noise in strain measurement.

Under different noise levels, the errors of displacement reconstruction are evaluated by relative differences between the exact results of direct FEM and the reconstructed results of iFEM. The relative difference is represented by the root mean square defined as

(13) $$\mathrm{RMS}=\sqrt{\frac{\sum_{i=1}^{N}{\left(\frac{w_{i}^{\mathrm{iFEM}}-w_{i}^{\mathrm{FEM}}}{\bar{\left|w^{\mathrm{FEM}}\right|}}\right)}^{2}}{N}}$$

where $w_{i}^{\mathrm{iFEM}}$ and $w_{i}^{\mathrm{FEM}}$ represent the reconstructed displacement from iFEM and the exact displacement from direct FEM, respectively; *i* and *N* are the index and number of iFEM nodes, respectively; $\bar{\left|w^{\mathrm{FEM}}\right|}$ is the mean of the absolute value of the FEM-based displacements. Figure 7 presents the variation of the relative difference subject to different noise levels. Increased errors of displacement reconstruction along with increasing noise level, from 0 to 10%, can be clearly seen. It should be noted that the reconstruction errors do not vanish corresponding to a noise level of 0%, because the displacements are reconstructed and thus will not be perfectly consistent with the exact displacements as computed directly. However, the reconstruction errors remain significantly low within a wide range of noise levels up to a high level of 10%. As an example, the maximum reconstruction error corresponding to noise level of 8% is less than 2.5%, as can be seen in Figure 7.

### 3.3. Damage Identification without Measurement Noise

Based on reconstructed vibration displacements using iFEM, the PE approach is then enabled to construct DI as expressed in Equation (11), relying on a finite difference scheme. As shown in Figure 5a–c, an inspection region is selected on the laminate surface within which the damage identification results are shown. The region is selected to exclude the influence of excitation on the identification results.

#### 3.3.1. Identification of Single Delamination Zones

**Figure 8:**
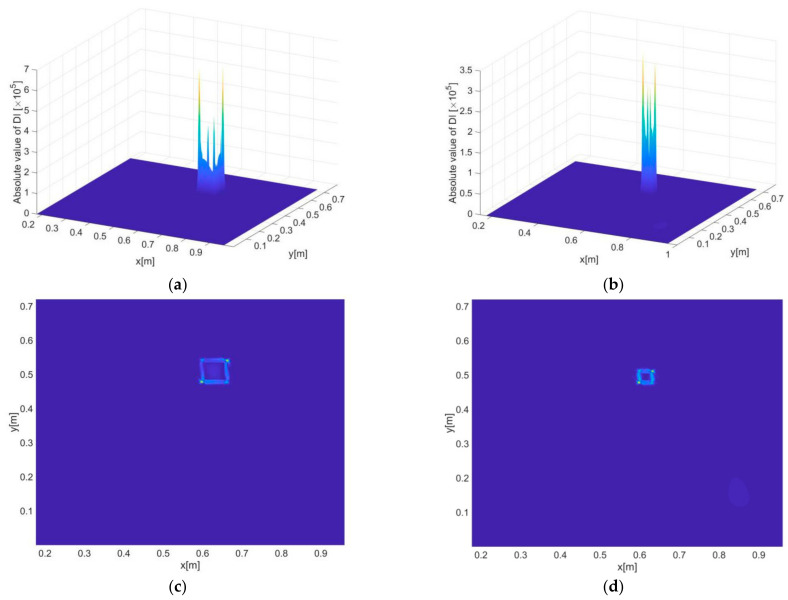

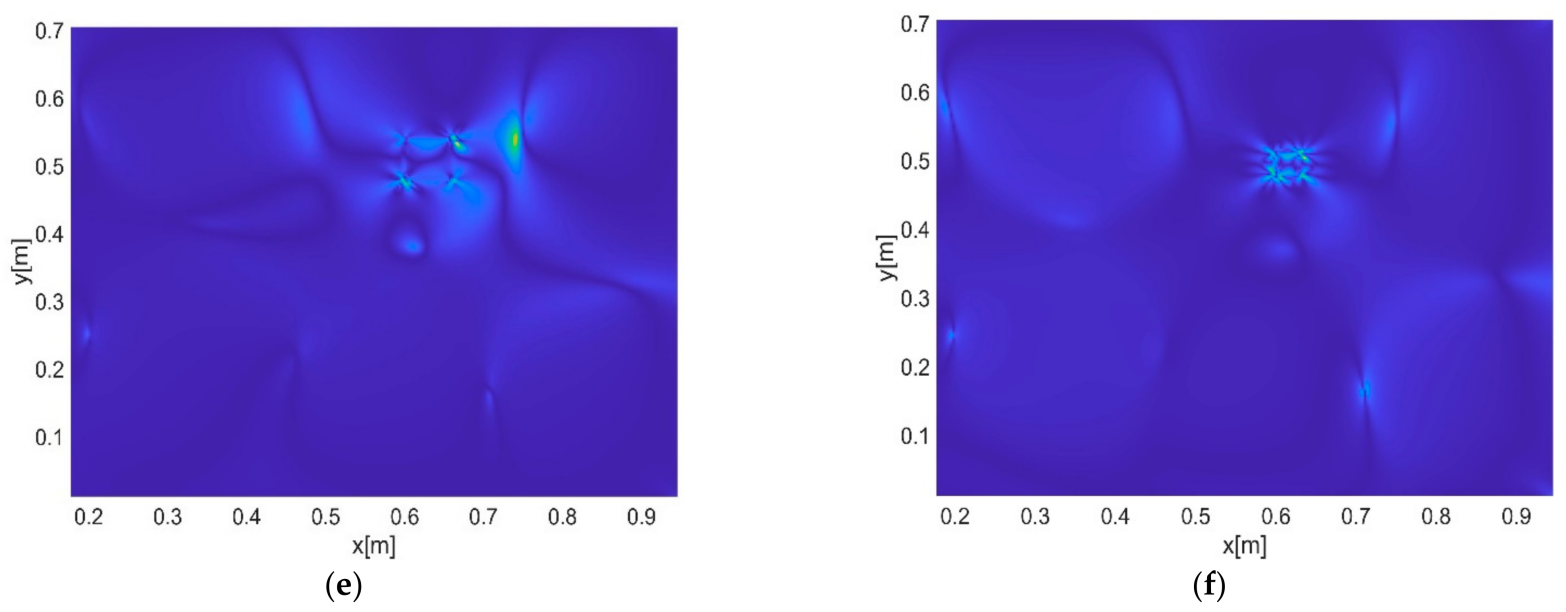
Constructed damage indexes (Dis) of the iFEM-PE method across the inspection region, corresponding to (**a**,**b**) damage scenario A, and (**c**,**d**) damage scenario B, respectively. (**a**,**c**) and (**b**,**d**) are the two- and three-dimensional views of DI, respectively. (**e**,**f**) present the damage identification results of scenario A and B, respectively, by using the method developed in [26].

Figure 8a–d present the delamination detection results (i.e., DI constructed by the iFEM-PE method) for scenario A and B, where both two- and three-dimensional presentations of DI are provided. It can be seen that the constructed DI is not only able to indicate the positions but also the exact shapes of the delaminations with both large and small sizes. The high sensitivity of DI to damage resides in its inclusion of high order derivatives of displacements that exhibit maximum singularities at the boundaries of damaged zones, and thus the shapes of damage can be precisely depicted by the DIs. Another reason for the high sensitivity is that the DI values at intact regions are strictly zero, as guaranteed by dynamic equilibrium conditions.

For comparison, the DI constructed in [26] is also used to identify the damaged zone, with results as shown in Figure 8e,f. It can be seen that while the DI shows certain sensitivity to the damage, the accuracy of damage identification is much lower than that of the iFEM-PE method. On the one hand, this is due to the DI seems only sensitive to the corners of the damaged zone and is unable to give exact information of damage boundaries. On the other hand, obvious non-zero fluctuations are found at the undamaged areas mainly due to the difference between the tested and baseline strains (reconstructed by iFEM) in a global sense, which becomes significant under the current vibration state, where the spatial alterations of strains and displacements are much more complex than in a static state of structures. In Figure 8e, in particular, a highlighted area at the right side of the damaged zone can be seen. Most likely, this may trigger false alarms of damage occurrence in real applications.

#### 3.3.2. Identification of Multiple Delamination Zones

**Figure 9:**
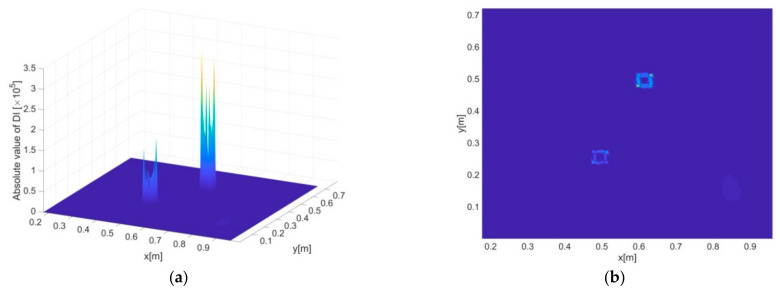
(**a**) Three- and (**b**) two-dimensional presentations of DI for delamination scenario C.

Based on the same excitation frequency and inverse element size, the two delamination zones included in scenario C are identified using DI, as shown in Figure 9a,b. As expected, the positions and shapes of the delaminations can be clearly indicated, although the singularity of DI associated with the lower left delamination in Figure 9b is less prominent compared to that associated with the upper right one. According to previous studies [32,34], the prominence of DI, as directly linked with detection accuracy, is influenced by multiple factors including damage severity, the distribution of internal bending moments, etc. For instance, it can be imagined that damage at locations with minimal internal bending moments (an extreme case is a damaged structure subject to no deformations, i.e., without any loads no matter static or dynamic) will be extremely difficult, or even impossible, to identify. However, relevant studies are beyond the scope of this work.

## 4. Parametric Discussion

### 4.1. Influence of Measurement Density

**Figure 10:**
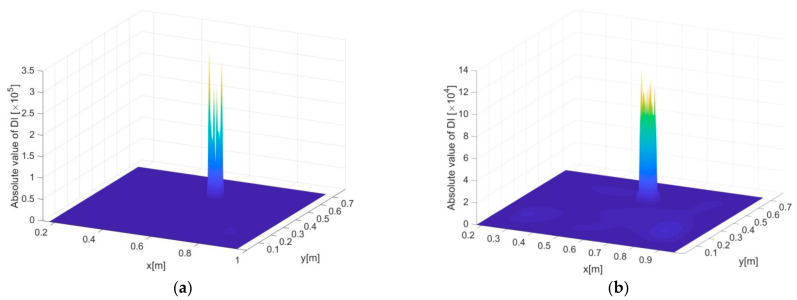

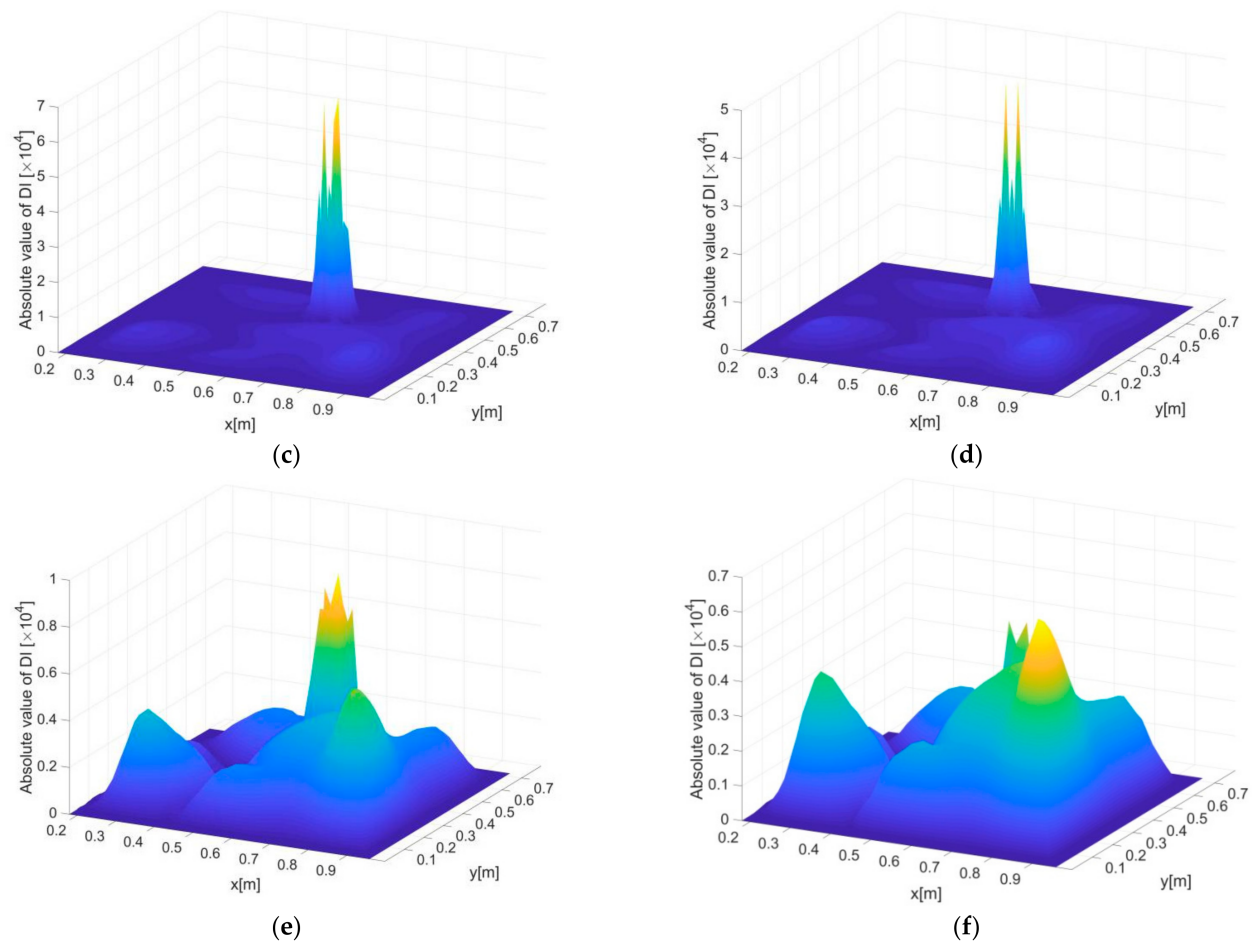
Delamination identification results using vibration displacements reconstructed by iFEM, subjected to an element size of (**a**) 3, (**b**) 6, (**c**) 12, (**d**) 15, (**e**) 24 and (**f**) 30 mm, respectively.

Of paramount importance is the measurement density directly associated with the number of sensors in real applications. In this section, damage scenario B, i.e., a single delamination with relatively small size, is identified by using different sizes of the inverse elements, recognizing that the measurement density decreases linearly with enlarged element sizes. Figure 10a–f show the DI constructed using the iFEM-PE method subject to element sizes of 3, 6, 12, 15, 24, and 30 mm, respectively. In general, detection accuracy degrades with enlarged element size. However, the overall accuracy is still considered high, i.e., the protrusion of DI is prominent at the delamination zone up to a large extent of elongation of the element size, e.g., 15 mm, as in Figure 10d. In Figure 10e, corresponding to inverse element size of 24 mm, the delamination can still be clearly revealed by DI. However, an obvious accuracy decrease starts to appear compared with the cases under smaller element sizes, by seeing that the region of DI protrusion is much broader than the actual delamination region, and the fluctuations of DI at the intact region become serious. In Figure 10f, corresponding to element size of 30 mm, the delamination can no longer be identified from DI distribution.

The main reason for the degraded detection accuracy is that the reconstruction errors of the vibration displacements increase with the enlargement of the inverse elements. While the accuracy can still be acceptable for pure displacement reconstruction, the accuracy of damage identification is unacceptable because of amplified noise influence due to the high-order derivatives in DI. Another reason resides in the enlarged distance between the difference points of the PE approach, which is consistent with the inverse element size. The difference points of PE should be sufficiently dense so as to reveal the fluctuations of vibration deflections. Otherwise, the finite difference scheme is not able to calculate DI values precisely because of too large truncation errors [32,34].

### 4.2. Influence of Measurement Noise

It has been demonstrated that the iFEM-PE method can be implemented effectively, independent of the measurement of vibration displacements, since strain data can be accurately transformed into displacements by using iFEM. This section focuses on the demonstration of the stronger noise immunity of the iFEM-PE method compared with the original PE approach. To do so, the delamination is identified by using displacements both obtained from direct FEM and reconstructed by iFEM. For FEM-based displacements, measurement noise is introduced according to an equation analogous to Equation (6), i.e., $w^{\mathrm{noisy}}=w^{\mathrm{exact}}\left(1+\Delta r\right)$, where $w^{\mathrm{noisy}}$ and $w^{\mathrm{exact}}$ are the noisy and exact displacements, respectively. In the following study, the noise introduced in the FEM-based displacements and the strains used by iFEM are with the same levels.

**Figure 11:**
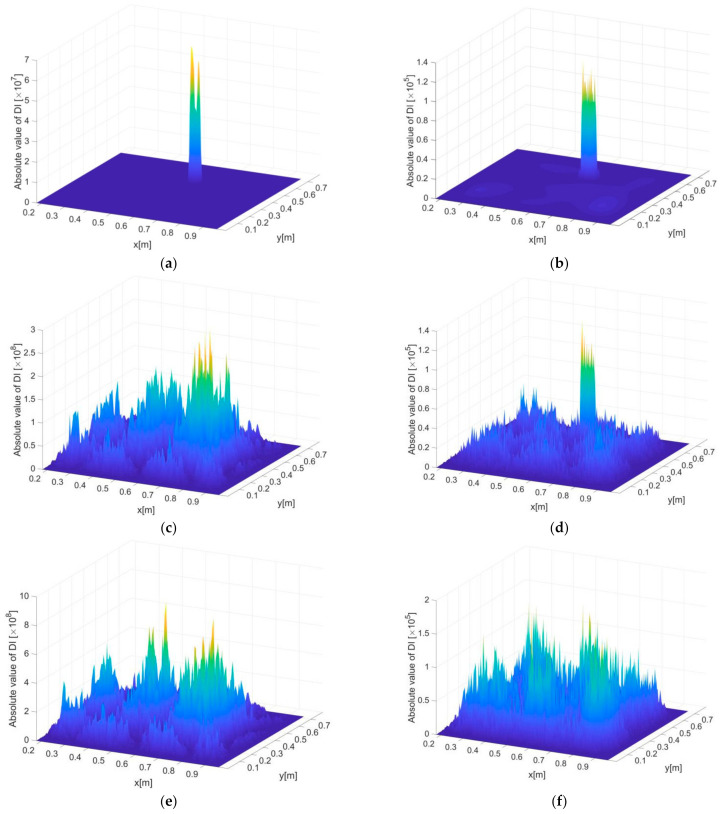
DI constructed based on the displacements computed by FEM and iFEM with (**a**,**b**) 0% (**c**,**d**) 1% and (**e**,**f**) 5% noise in level, subjected to measurement inter-distance of 6 mm.

Figure 11a–f show the delamination detection results under noise levels of 0, 1 and 3%, where Figure 11a,c,e present the FEM-based results and Figure 11b,d,f present the iFEM-based results. The distance between adjacent difference points of PE is 6 mm. Note that the element size of direct FEM is constant to be 1.5 mm as introduced before, so for FEM-based results the difference points are just selected from a constant number of FE nodes according to a given point distance, whereas for iFEM-based results, the point distance is consistent with the size of the inverse elements, meaning that increased point distance corresponds to enlarged inverse elements, reduced density of strain measurement and thus increased reconstruction errors.

Without noise influence, it can be seen by comparing Figure 11a,b that the FEM-based DIs are more accurate in damage detection (i.e., more localized DI protrusion corresponding to the delamiantion zone) than the iFEM-based ones, which is reasonable since direct FEM involves no reconstruction errors. Unfortunately, the FEM-based DIs fail to indicate the delamination under a noise level of 1%, as shown in Figure 11c, whereas stronger noise resistance of the iFEM-based DIs can be clearly seen in Figure 11d, in which the delamination is clearly localized. Under a noise level of 3%, both the FEM- and iFEM-based DIs are contaminated too seriously by noise to reveal the location of the delamination.

As explained, DI includes fourth order derivatives of vibration displacements, which makes it particularly sensitive to noise influence because even a slight degree of noise in the displacements can be amplified drastically through the implementation of the finite difference scheme. However, the iFEM-based DIs have apparently improved noise immunity because measurement noise is involved in strains rather than displacements. A straightforward interpretation is that, considering the strains are two-order derivatives of displacements in an equivalent sense, the derivation of strains only needs to be performed twice to construct the fourth-order derivatives of displacements (although in the iFEM-PE method the strains are first transformed into displacements and then differentiated). This means the noise is only amplified twice in the DI construction process. However, for FEM-based DIs constructed by four derivations of the measured displacements, the noise involved is also amplified four times during the DI construction process.

**Figure 12:**
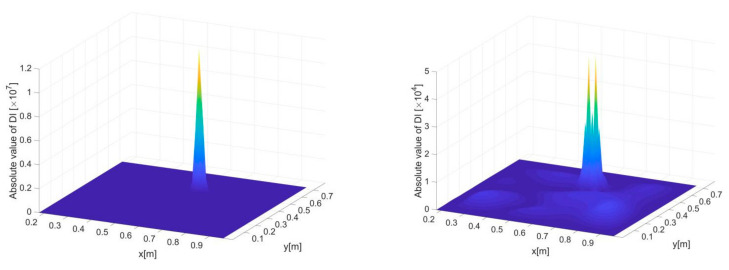

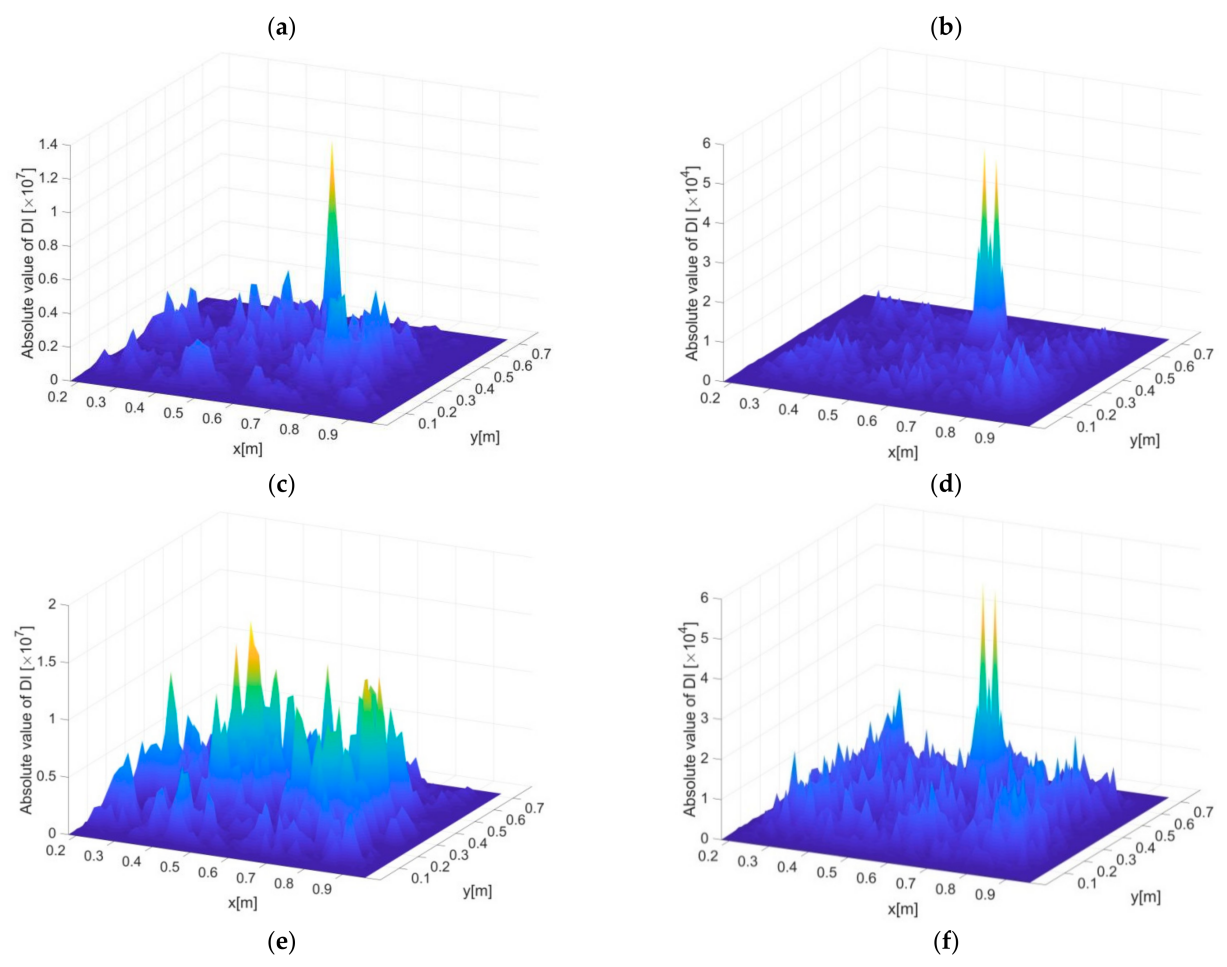
DI constructed based on the displacements computed by FEM and iFEM with (**a**,**b**) 0% (**c**,**d**) 1% and (**e**,**f**) 5% noise in level, subjected to measurement inter-distance of 15 mm.

Subjected to the similar levels of noise influence as in Figure 11, the point distance (i.e., size of inverse elements for iFEM) is further enlarged to be 15 mm, giving rise to delamination detection results as shown in Figure 12a–f, from which several interesting points can be seen. First, it is reasonable that without noise influence, the accuracy of detection based on both FEM and iFEM decreases, by comparing Figure 11a,b and Figure 12a,b. Such a decrease is associated with reduced finite difference accuracy. More interestingly, improved noise immunity associated with the increase in inverse element size is found in the noisy cases. Under noise levels of 1%, the accuracy of FEM-based DIs is significantly enhanced, as seen by comparing Figure 11c and Figure 12c, although some false peaks in the intact regions exist in Figure 12c and may affect the reliability of damage detection. More accurate results are seen in Figure 12d, where the delamination is precisely revealed by the iFEM-based DIs, showing remarkable accuracy, even comparable to the noise-free case in Figure 12b. Under a noise level of 3%, the FEM-based DIs are no longer able to identify damage because of severe noise interference (see Figure 12e), whereas the iFEM-based DIs still have a satisfactory capacity for delamination localization, as seen in Figure 12f. It should be noted that the current identification results are not facilitated by any de-noising treatment for accuracy improvement.

Besides the previous explanation for stronger noise resistance of the iFEM-based results compared to that of the FEM-based results, the further enhanced noise immunity of the iFEM-based results subject to increased inverse element size, as seen by comparing Figure 11 and Figure 12, is attributed to the fact that the noise amplification effect of the high-order derivatives in DI is significant under small point distances. So, smaller inverse element sizes will lead to larger noise, accumulated by finite difference, that overwhelms the damage feature in DI. When the inverse element size is enlarged, the noise will be increasingly suppressed to enable the recognition of damage features. Nevertheless, the inverse element size should be kept below a reasonable value for the toleration of truncation errors of finite difference. Otherwise, the detection accuracy will fade rapidly even under noise-free conditions.

### 4.3. AD-NI Analysis

To facilitate the illustration of the comprehensive influence of different factors on detection accuracy, two parameters, i.e., accuracy of difference (AD) and noise influence (NI), are used. The meanings of AD and NI are briefed here, as a detailed definition can be found in [33]. Accuracy of difference (AD) represents the accuracy of the finite difference scheme under noise-free conditions. Along the structural surface, an equivalent estimation region is set to cover the damaged zone, and AD is expressed as

(14) $$AD=\frac{r}{r_{c}}$$

where *r* is the ratio of the maximum (absolute value of) DI outside the estimation region to that within the region. Thus, *r* is able to indicate how prominent the DI values at the damaged zones compared with those at the intact regions, i.e., smaller *r* implies higher accuracy of difference. The threshold of *r* is set to be $r_{c}$, below which the accuracy of difference is acceptable. The acceptable range of AD is normalized according to Equation (14) to be [0, 1].

NI represents the noise influence, which is associate with correlation coefficient defined as Ξ [34]. Ξ indicates the extent of similarity of DI distributions with and without noise influence. The value of Ξ ranges from 0 to 1, where a minimal value close to 0 indicates extreme discrepancy between the exact and noisy DI distributions, whereas 1 indicates complete coincidence of the exact and noisy DIs, interpreted as perfect noise resistance with zero signal disturbance due to noise. NI is then defined as

(15) $$\mathrm{NI}=\frac{1-\Xi }{1-\Xi_{c}}$$

where $\Xi_{c}$ is a threshold. According to Equation (15), the acceptable range of NI is also normalized within [0,1], similar with that of AD.

**Figure 13:**
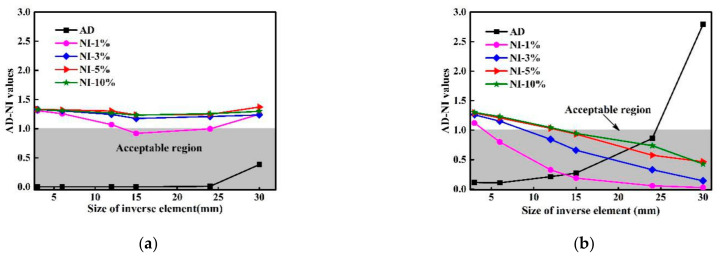
AD–NI curves for the (**a**) FEM-based and (**b**) iFEM-based damage identification results.

By setting both $r_{c}$ and $\Xi_{c}$ to be 0.75, Figure 13a,b present the FEM- and iFEM-based AD–NI curves by taking into account the influence of point distance and noise level. A general principle is that with respect to a given point distance, damage is deemed to be identifiable when both AD and NI fall into the acceptable region. Specifically, the FEM-based AD curve in Figure 13a shows much smaller magnitudes than the iFEM-based curve in Figure 13b, indicating higher accuracy of difference. Such an observation implies increasing reconstruction errors of iFEM with enlarged size of inverse elements. However, it is of extreme difficulty for the FEM-based results to identify damage successfully because of its high sensitivity to noise influence. As shown in Figure 13a, delamination is only possible to detect under a point distance of 15 mm and under a noise level of 1%, the DI distribution corresponding to which is shown in Figure 12c. For the iFEM-based results, it is interesting to see that a number of options can be made for delamination detection. First, with inverse element size of 3 mm, detection accuracy is low due to severe noise influence (by seeing that all NI values are beyond the acceptable region), although the accuracy of difference represented by AD is good. With an element size of 6 mm, delamination can be detected with noise level of 1%, whereas further increased noise levels lead to unacceptable detection accuracy. With further increased inverse element sizes, noise immunity is enhanced continuously. Corresponding to an element size of 24 mm, in particular, all NI-AD pairs are within the acceptable region, indicating that the delamination can be identified in all cases even up to a noise level of 10% in the measured strains. With an element size of 30 mm, delamination can no longer be revealed because of unacceptable AD, although the noise immunity is considerably strong, as indicated by the NI values.

## 5. Delamination Identification under Simulated Structural Operational States

In this section, multiple delamination zones in the CFRP laminate in scenario C, as shown in Figure 5c, are identified using the iFEM-PE method, by considering various requirements in structural operational states. Additional treatments, including uni-axial strain measurement and hybrid data fusion, are performed to achieve optimal detection results.

### 5.1. Uni-Axial Strain Measurement

**Figure 14:**
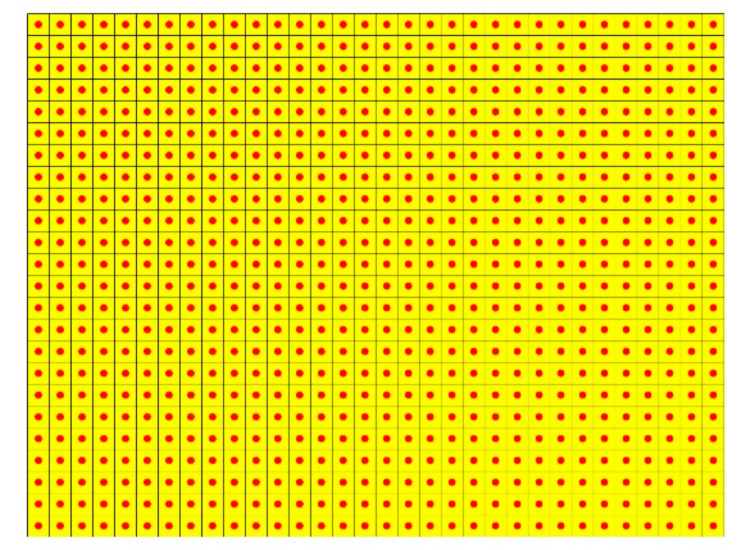
Actual positions of strain measurement points and the inverse elements drawn on the laminate surface.

**Figure 15:**
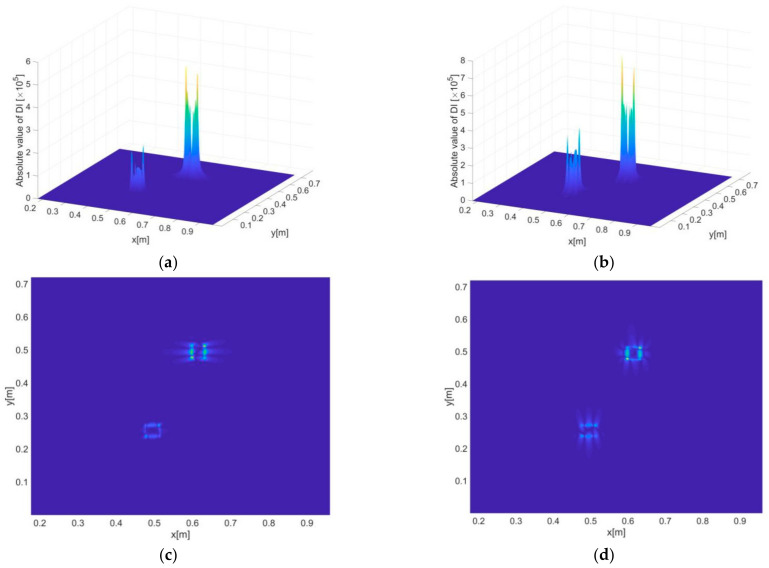
Delamination identification results of scenario C by using uni-axial strain measurement: (**a**) and (**c**) display the three- and two-dimensional views of detection results using strains in *x* direction, respectively, whereas (**b**) and (**d**) display the three- and two-dimensional views of detection results using strains in *y* direction, respectively.

As introduced, the strain measurement density is adjusted in steps to reduce the amount of sensors. That is, the in-plain shear strain, $\gamma_{xy}$ in Equation (3), is first omitted to prevent the usage of strain rosettes, and single side strain measurement is then adopted to further reduce the sensor amount by half. The measurement density is further reduced by noticing that enlarged element size is able to enhance noise resistance. In this section, uni-axial strain measurement is adopted instead of bi-axial strain measurement, by considering that uni-axial strains are able to capture sufficient vibration information associate with damage features. Figure 14 presents the actual positions of strain measurement points and the inverse elements on the laminate, and Figure 15a–d present the two- and three-dimensional views of the noise-free DI distributions subject to uni-axial strain measurement with inverse element size of 3 mm. It can be seen that highly accurate results are obtained based on strains in both the *x* (in Figure 15a,c) and *y* directions (in Figure 15b–d). It should be admitted that damage information based on uni-axial strain measurement is slightly incomplete compared to that based on bi-axial strain measurement (as in Figure 9a,b). For example, the upper and lower boundaries of the upper-right delamination are not clearly revealed by only using *x*-direction strains, as shown in Figure 15c, whereas the left and right boundaries of the lower-left delamination are not clear by using *y*-direction strains, as shown in Figure 15d. However, the features associated with the positions and sizes of the delamination zones are still prominent in the figures.

### 5.2. Hybrid Data Fusion

One main restriction in experiments is that the damage locations and severity cannot be known in advance, so it is basically impossible to select an optimal single-value excitation frequency that is most sensitive to all possible damage. Moreover, harmonic excitation normally involves additional experimental set-up, causing unnecessary complexity of the iFEM-PE method to be applied in practice. Considering that broad band vibrations occur naturally under structural operational state, a series of harmonic responses can be extracted from the broad band responses to generate a number of independent detection results, which can be fused together to produce an ultimate result with enhanced accuracy and reliability.

Given a DI value obtained under K different measurement circumstances (e.g., different frequencies, element sizes, etc.), denoted by ${\mathrm{DI}}_{1},\cdots {\mathrm{DI}}_{K}$, the hybrid fusion algorithm is defined as

(16) $${\mathrm{DI}}_{hybrid}={\mathrm{DI}}_{\mathrm{arithmetic}}\cap {\mathrm{DI}}_{\mathrm{geometric}}$$

where

(17) $${\mathrm{DI}}_{\mathrm{arithmetic}}=\frac{1}{K}\sum_{i=1}^{K}{\mathrm{DI}}_{i}$$

and

$${\mathrm{DI}}_{\mathrm{geometric}}=\sqrt[{K}]{{\mathrm{DI}}_{1}\times {\mathrm{DI}}_{2}\times \cdots \times {\mathrm{DI}}_{K}}$$

(18

${\mathrm{DI}}_{hybrid}$ is the ultimate field value upon hybrid fusion. Development of such a hybrid fusion algorithm is motivated by the incentive to reap the merits of individual data fusion schemes including the arithmetic and geometricfusion, so as to maximize the fusion efficiency with limited information sources.

### 5.3. Delamination Identification Results

At last, multiple delamination zones in scenario C are identified, relying on uni-axial strains in *x* directions. The inverse element size is selected to be 20 mm, corresponding to a measurement density that can be easily achieved by existing strain sensor network, and the noise level is set to be 5%, which is considered significant even in practical measurement. Dynamic responses are assumed to be extracted from a wide frequency band of 50–200 Hz with an uniform interval of 10 Hz, leading to sixteen independent responses subject to a frequency series of 50, 60, …, 200 Hz. In addition, for each frequency, the strain measurement is averaged three times, which is a common method of signal treatment that is frequently utilized in practice.

**Figure 16:**
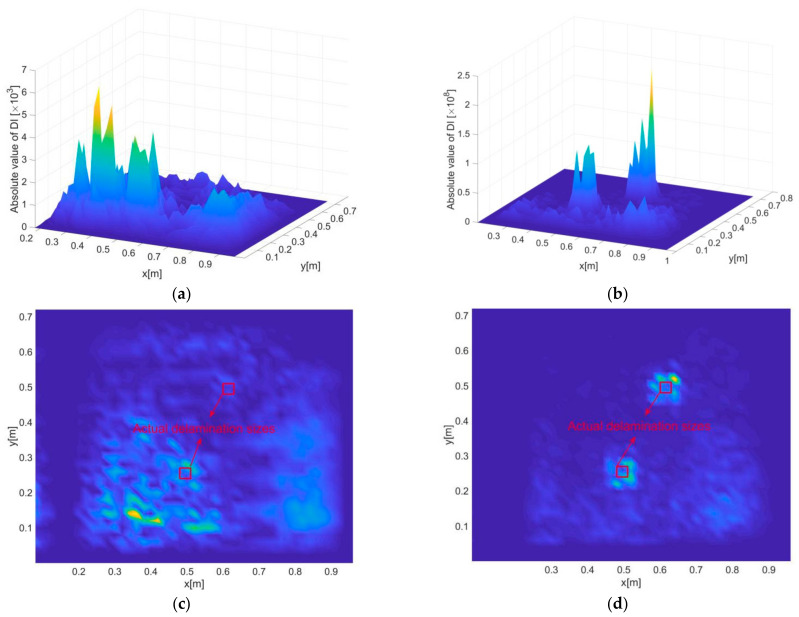
Delamination detection results for scenario C, where (**a**) and (**c**) present the three- and two-dimensional views of the results under excitation frequency of 100 Hz, and (**b**) and (**d**) present the three- and two-dimensional views of the results subject to hybrid data fusion.

The delamination identification results subject to a single frequency of 50 Hz are shown in Figure 16a,c. Under the given inverse element size and noise level, the DIs are not able to reveal either of the two delamination zones because of the too disrupted DI distribution. The results based on data fusion no doubt have a largely improved accuracy, as shown in Figure 16b,d. While the identified sizes of the delaminations are larger than the actual sizes (due to relatively big point distances), the revealed positions and overall shapes of the delaminations are still clear and accurate. Finally, it should be emphasized that the purpose of data fusion is to maximize the reliability and robustness of damage identification without prior knowledge of damage and excitation, although it is possible under some circumstances that certain particular single-value frequencies may even lead to better results than using data fusion.

## 6. Conclusions

According to the numerical results of delamination identification in a CFRF laminate, the apparent advantages of the iFEM-PE method have been demonstrated. The independence of vibration displacement measurement can be seen as a key merit of the method that enables online damage identification by using most existing on-board SHM systems. On the other hand, the significant enhancement of noise immunity, benefiting from direct strain measurement, is crucial for the method to adapt to structural operational states, under which large interference of measurement noise is unpreventable.

The sensor number can be largely reduced by simplifying the sensor layout strategy. In the current study, neglection of in-plane shear strains, single-side and uni-axial strain measurement are proven to be three effective ways of controlling the sensor number, without apparently sacrificing the accuracy of displacement reconstruction and damage identification. Although large measurement density is considered able to include richer information of structural vibration, at least two merits are obvious by reducing the measurement density, i.e., further reduced sensor number and increased noise immunity. The drawback of measurement density reduction (enlargement of inverse elements) is that the noise-free accuracy of damage detection degrades gradually because of increased reconstruction errors, which, when beyond a certain extent, will lead to ultimate failure of damage detection, regardless of the improved noise resistance. Such a phenomenon can be clearly observed from the AD–NI curves. Therefore, sophisticated selection of proper measurement density, by taking into account vibration frequencies, structural properties, etc., is required in real applications to achieve optimal detection results. Nevertheless, a rule of thumb should be kept in mind that compared to deformation reconstruction, much denser measurement is needed for damage detection in order to capture the local signal disturbance associated with damage. Finally, hybrid data fusion is proven to be an effective scheme in highlighting damage features by processing detection results from a series of independent data sources. Relying on data fusion, multiple delamination zones in the CFRP laminate are identified successfully subject to a considerably high level of measurement noise, i.e., $\sigma \left(\Delta r\right)=5\%$.

Some interesting topics can be carried out in the future to facilitate the development of the iFEM-PE method. For example, the effectiveness of other forms of inverse element formulations in displacement reconstruction can be explored, which may provide additional gains in detection accuracy; Furthermore, wide band excitation can be applied to generate more realistic vibration responses that are common in engineering practice. Data fusion can be applied to process a large number of extracted narrow band responses to produce precise and reliable detection results.

## Figures and Tables

**Table 1:** Mechanical properties of the CFRP lamina.

Young’s Modulus [GPa] (E1/E2/E3)	Shear Modulus [GPa] (G12/G13/G23)	Poisson’s Ratio (v12/v13/v23)	$\mathrm{Density}\;[{\mathrm{kg}/m}^{3}]$
157.9/9.6/9.6	5.9/5.9/3.2	0.32/0.32/0.49	1620

## Data Availability

Data sharing not applicable. No new data were created or analyzed in this study. Data sharing is not applicable to this article.
